# Neither the pan-immune-inflammation value nor the PILE score improved on APACHE-II or SOFA in a single-center cohort of critically ill older patients with acute myeloid leukemia

**DOI:** 10.3389/fmed.2026.1955731

**Published:** 2026-10-27

**Authors:** Ayse Muge Karcioglu, Selin Kucukyurt Kaya, Hatice Zeynep Atli, Emel Acar, Ahmet Kursad Gunes

**Affiliations:** 1 Department of Intensive Care, Ministry of Health Etlik City Hospital, Ankara, Türkiye; 2 Department of Hematology, Ministry of Health Etlik City Hospital, Ankara, Türkiye

**Keywords:** acute myeloid leukemia, APACHE-II, intensive care unit, older adults, overall survival, pan-immune-inflammation value, PILE score

## Abstract

**Background:**

Older patients with acute myeloid leukemia who require intensive care admission have high mortality, and decisions regarding treatment escalation lack objective guidance. Inflammation-based indices derived from routine blood counts are widely reported in ambulatory oncology, but whether they hold any value once a patient becomes critically ill remains unestablished. We investigated whether the pan-immune-inflammation value (PIV) and the PILE score predict intensive care mortality and whether they add anything to the severity scores already in use.

**Methods:**

In 75 patients aged 65 years or older admitted to a tertiary intensive care unit (ICU), both indices were computed at diagnosis and at ICU admission and compared with APACHE-II and SOFA scores. The primary endpoint was ICU mortality; overall survival was analyzed from diagnosis with delayed entry at ICU admission, and incremental value was assessed in nested logistic regression models.

**Results:**

ICU mortality was 72.0%. APACHE-II and SOFA discriminated ICU mortality (area under the curve 0.778, 95% CI 0.646–0.892 and 0.758, 0.640–0.865), each significantly better than either PIV score on pairwise testing. Neither PIV score discriminated, and only the ICU-admission PILE score reached nominal significance (0.647; *p* = 0.037). Neither index added an increment after correction for multiple comparisons, and no other count-based index performed better. Measured from diagnosis, a higher baseline PILE score was associated with shorter overall survival after adjustment for ELN risk, comorbidity, treatment, and disease status (hazard ratio 1.95, 95% CI 1.33–2.85), although this was not specific to PILE and did not persist once survival was calculated from ICU admission. With 54 deaths, the smallest area under the curve separable from 0.5 with 80% power was 0.681.

**Conclusion:**

An index calculated as the product of cellular counts is limited in its ability to stratify cytopenic patients: in nine patients, a neutrophil or monocyte count of zero drove PIV to zero, so the score classified the most complete marrow failure as the lowest inflammatory risk. Neither index improved upon existing ICU severity scores. Any prognostic role they hold more likely lies at diagnosis, while treatment intensity and the ceiling of care remain open questions.

## Introduction

Acute myeloid leukemia (AML) is the most common acute leukemia in adults and largely a disease of older age, with a median age at diagnosis of approximately 68 years (1). Survival is poor and worsens sharply with age: the 5-year survival rate is approximately a third overall but drops to single digits in the oldest patients (2). Several features associated with advanced age contribute to these outcomes, including a heavier comorbidity burden, poorer performance status, limited tolerance of intensive chemotherapy, and more frequent adverse cytogenetic and molecular findings (3).

Patients with hematological malignancies require intensive care for several reasons, including disease progression, infection, and complications of treatment. About a fifth of hospitalized patients with these cancers require an intensive care unit (ICU) stay (4), and mortality among them is high, reaching 56% in the unit and 72% in hospital in one large series (5). Among patients with hematological malignancies who received chemotherapy in the unit, ICU mortality was 42% and 1-year mortality 64% (6). In AML, the immediate threat is usually infection and acute organ failure, arising against a background of bone marrow failure (7, 8). Early and reliable risk assessment is therefore critical, both to identify deteriorating patients and to guide decisions regarding treatment intensity and goals of care. Severity scores such as APACHE-II, SOFA and SAPS II are widely used in critically ill patients with cancer (9–11), yet tools designed specifically for AML have been tested in only a handful of studies (12).

The pan-immune-inflammation value (PIV) is a composite marker derived from peripheral neutrophil, monocyte, platelet, and lymphocyte counts (13), and the PILE score adds the Eastern Cooperative Oncology Group (ECOG) performance status and the serum lactate dehydrogenase (LDH) to PIV to give a simple bedside index (14). Higher values of both indices have been associated with worse outcomes across a range of solid tumors (15). A smaller body of literature has demonstrated similar associations in hematological malignancies, including lymphoma (16) and a link between PIV and overall survival in AML (17). Whether either score retains prognostic value once a patient with AML is critically ill, and how these indices compare with severity scores already in use in the ICU, has not been examined.

We therefore evaluated patients aged 65 years or older with AML admitted to a tertiary intensive care unit. The primary objective was to determine whether PIV and PILE predict ICU mortality in this group, how they compare with APACHE-II and SOFA, and evaluate whether they provide incremental predictive value beyond these established systems. Overall survival was examined as a secondary endpoint.

## Materials and methods

### Study design and ethics

This was a single-center, retrospective cohort study approved by the Ethics Committee of Ministry of Health, Ankara Etlik City Hospital (approval number AEŞH-BADEK1-2025-287, dated 16 July 2025) and conducted in accordance with the principles of the Declaration of Helsinki. The committee waived the requirement for informed consent because of the retrospective design. Reporting follows the STROBE reporting guidelines for observational studies.

### Patients

We screened all patients admitted to the general ICU between 1 January 2023 and 1 January 2025, admitted either from the emergency department or from an inpatient ward. Eligibility criteria included an age of 65 years or older and a confirmed diagnosis of AML. The threshold of 65 years was chosen because it falls just below the median age at diagnosis and therefore captures the majority of the AML population, in whom comorbidity and performance status weigh most heavily on treatment decisions. Patients whose leukemia had been diagnosed at another hospital were excluded, as the absence of diagnostic records precluded computation of baseline scores. For patients with multiple ICU admissions, only the first admission following AML diagnosis was analyzed. Figure 1 shows the flow of patients through the study.

**Figure 1:**
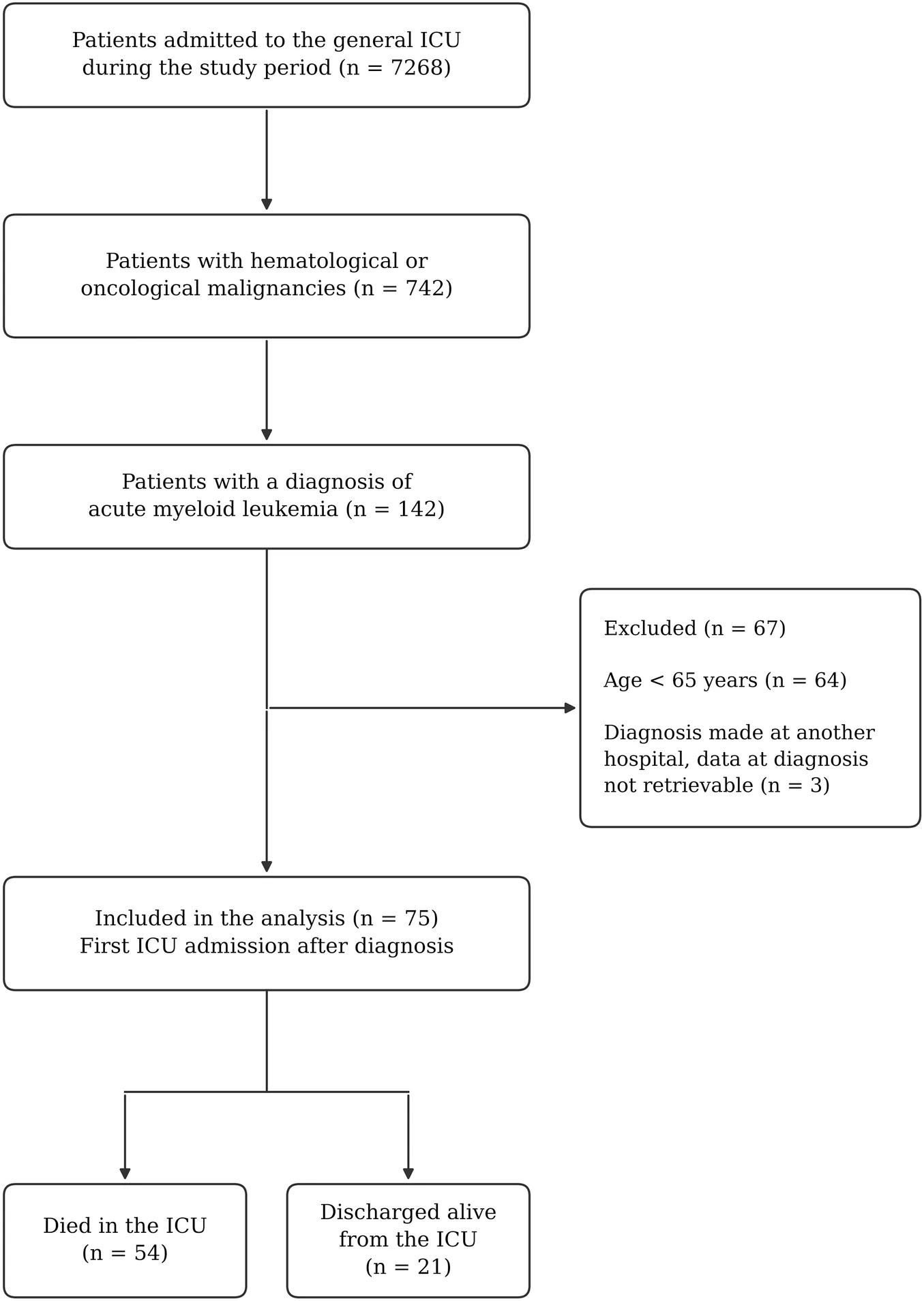
Flow of patients through the study. AML, acute myeloid leukemia; ICU, intensive care unit.

### Data collection

For each patient, we recorded age, sex, comorbidities, date of AML diagnosis (taken as the date of bone marrow aspiration and biopsy), AML subtype, ELN 2022 risk category, Charlson Comorbidity Index, disease status at ICU admission (remission or active disease), receipt of cytoreductive therapy and of allogeneic hematopoietic stem cell transplantation, the complete blood cell count and serum LDH at diagnosis, bone marrow blast percentage, and any preceding myelodysplastic or myeloproliferative disorder. Performance status was recorded twice. The first score was based on documentation at the time of diagnosis. For the second, we excluded the 14 days before ICU admission, because a score assigned during that period would capture the acute deterioration leading to admission rather than the patient's usual functional state, and we recorded the most recent score documented before that 2-week window. Both score were available, for every patient included. From routine laboratory tests obtained at ICU admission, we obtained complete blood cell count, biochemistry, blood gas, and acute-phase reactants, and calculated the APACHE-II and SOFA scores. We also recorded the events of the ICU stay: pneumonia, sepsis, acute kidney injury, soft-tissue infection, invasive mechanical ventilation, extubation, vasopressor support and its duration, transfusion counts, the mode of ICU discharge and ICU mortality. These events arise during the critical illness and are shaped by it, so they were treated as descriptors of the clinical course rather than as predictors of mortality.

### Endpoints

The primary endpoint was ICU mortality and the secondary endpoint was overall survival. Patients became eligible only upon admission to the ICU, so calculating survival from the date of diagnosis would credit each of them with the interval already survived before the study began. To prevent this, overall survival was analyzed on the time scale from diagnosis with delayed entry, each patient joining the risk set on the day of ICU admission, and the analysis was subsequently repeated using ICU admission as the time origin. Death from any cause was the event of interest. Patients still alive were censored at the closing follow-up date of 1 June 2026, yielding 72 deaths and three censored observations.

### PIV and PILE scores

PIV was calculated as neutrophil × platelet × monocyte/lymphocyte (each in × 10^9^/L), separately from the blood counts at diagnosis and at ICU admission. The PILE score summed three components: one point for a PIV at or above its own time-point median (0 below), one point for LDH at or above the laboratory upper limit of normal of 223 U/L (0 below), and one point for an ECOG performance status of 2 or more (0 below). The resulting 0–3 score was stratified as low risk (0–1) or high risk (2–3). Each score drew its PIV threshold from the median at the time point, at which the blood count was taken, because counts obtained at diagnosis and those obtained during critical illness describe different clinical states, and one cut-off applied to both would judge them by a yardstick that fits neither. This definition ties the threshold to our own cohort, so we repeated the analysis using a single median pooled across the two time points. PIV entered the regression models as a log-transformed continuous variable, with effect estimates expressed per standard deviation, and was dichotomized only for group comparisons. APACHE-II and SOFA were treated as continuous variables throughout, in keeping with their conventional use.

### Statistical analyses

Descriptive and comparative analyses were conducted using IBM SPSS Statistics version 27. The DeLong test, bootstrap procedures, and survival models were run in R, using the pROC package for the curve comparisons. Normality was assessed using skewness and kurtosis; values within ±3 were considered sufficient to support parametric analysis. Continuous variables are summarized as mean ± SD or median (interquartile range), depending on their distribution, and categorical variables as counts and percentages. Two groups were compared using the independent-samples *t*-test or the Mann–Whitney *U*-test, and categorical variables were compared using the Pearson chi-square test, or Fisher's exact test when an expected cell count was below five. Correlations between the scores and the length of ICU stay were assessed with Spearman's rank correlation.

Discrimination of ICU mortality was assessed by receiver operating characteristic (ROC) analysis, reporting the area under the curve (AUC) with its 95% confidence interval and, for scores that discriminated significantly, the Youden index cut-off with its sensitivity and specificity. Curves were compared pairwise using the DeLong test. To assess whether the inflammatory indices provided incremental prognostic value beyond the established severity scores, each index was added, in turn, to APACHE-II and SOFA in nested logistic regression models. The nested models were compared using the likelihood ratio test, and the change in the area under the curve was estimated with a 95% confidence interval based on 3,000 bootstrap resamples. Because four such comparisons were made, a Bonferroni-corrected threshold of *p* < 0.0125 was applied. Two exploratory analyses examined the discrimination of ICU mortality by each blood cell count component at ICU admission and by each score within the 2022 ELN intermediate- and adverse-risk groups; for the subgroup analyses, a Bonferroni-corrected threshold covering all 16 comparisons was applied.

Overall survival was modeled using Cox proportional-hazards regression with delayed entry on the time scale described above, and the same models were refitted with ICU admission as the time origin. The association between the baseline PILE score and survival was adjusted for ELN 2022 adverse risk, the Charlson Comorbidity Index, intensive induction chemotherapy, and active disease at ICU admission. Where PIV entered a model, it was log-transformed after adding a constant equal to half the smallest positive value observed at that time point, because a neutrophil or monocyte count of zero resulted in nine patients with a PIV of zero at ICU admission. Survival curves were estimated using the Kaplan–Meier method from ICU admission and compared using the log-rank test, since under delayed entry the number of patients at risk at any point measured from diagnosis was too small to support a stable curve. A two-sided *p* < 0.05 was taken as significant.

No *a priori* sample size calculation was performed, as the cohort comprised all eligible patients admitted during the study period. To contextualize the negative findings, we calculated the smallest effect sizes the cohort could have detected with 80% statistical power at a two-sided alpha of 0.05, using the observed numbers of patients and events and, for the comparisons of correlated areas under the curve, the covariance obtained from the DeLong procedure.

## Results

Between 1 January 2023 and 1 January 2025, 7,268 patients were admitted to the general ICU, 742 of whom had a hematological or oncological malignancy. Of these, 142 had a diagnosis of AML. A total of 64 were younger than 65 years, and 3 had been diagnosed at another hospital, so their data at the time of diagnosis could not be retrieved; both groups were excluded. The remaining 75 patients formed the study cohort, and for each of them the first ICU admission following AML diagnosis was analyzed (Figure 1).

The cohort comprised 75 patients with a mean age of 75.1 ± 6.2 years, of whom 68.0% were men. Adverse-risk disease by the ELN 2022 classification was present in 56.3% of the 71 patients for whom risk could be assigned, and the mean Charlson comorbidity index was 6.4 ± 2.0. By the French–American–British (FAB) classification, the M0–M2 group was the most common subtype (44.0%), followed by M4–M5 (18.7%); subtype was not recorded in 21.3% of patients, and the marrow blast percentage at diagnosis was a median of 50% (IQR 20–80). AML was *de novo* in 54.7%, while the remainder followed a preceding hematological disorder, most often myelodysplastic syndrome (36.0%). An ECOG performance status of 2 or more was recorded in 66.7% of patients at diagnosis and in 88.0% at the last assessment before ICU admission. Active leukemia was present in 69.3% at the time of ICU admission, and 8.0% had received intensive induction chemotherapy. Respiratory failure was the leading reason for admission (61.3%). A total of 54 patients (72.0%) died in the ICU. None of these baseline characteristics differed significantly between those who died and those discharged alive (Table 1).

**Table 1:** Baseline characteristics by ICU mortality.

Characteristic	Total (*n* = 75)	Died (*n* = 54)	Discharged (*n* = 21)	*p*
Age, years, mean ± SD	75.1 ± 6.2	75.5 ± 5.5	74.1 ± 7.8	0.456
Male sex	51 (68.0)	40 (74.1)	11 (52.4)	0.071
ELN 2022 risk				0.593
Favorable	2 (2.8)	2 (3.8)	0 (0)	
Intermediate	29 (40.8)	22 (42.3)	7 (36.8)	
Adverse	40 (56.3)	28 (53.8)	12 (63.2)	
Charlson comorbidity index, mean ± SD	6.4 ± 2.0	6.6 ± 2.0	6.1 ± 2.0	0.384
ECOG ≥ 2 at diagnosis	50 (66.7)	35 (64.8)	15 (71.4)	0.585
ECOG ≥ 2 before ICU admission	66 (88.0)	50 (92.6)	16 (76.2)	0.106
Active disease at ICU admission	52 (69.3)	37 (68.5)	15 (71.4)	0.806
Intensive induction chemotherapy	6 (8.0)	4 (7.4)	2 (9.5)	1.000
Respiratory failure as reason for admission	46 (61.3)	34 (63.0)	12 (57.1)	0.640

ECOG, Eastern Cooperative Oncology Group; ELN, European LeukemiaNet.

Data are *n* (%) unless stated. ELN 2022 risk could be assigned in 71 patients (52 who died and 19 discharged); the percentages and the *p*-value refer to this classified subgroup, and the *p-*value compares the three risk categories. Values are compared by the independent-samples *t*-test with equal variances not assumed, the Pearson chi-square test, or Fisher's exact test as appropriate.

The APACHE-II and SOFA scores were higher in patients who died (29.3 ± 7.1 vs. 22.6 ± 5.4 and 9.8 ± 2.9 vs. 7.0 ± 2.6; both *p* < 0.001). Serum LDH was higher at diagnosis and at ICU admission in non-survivors, as were admission C-reactive protein (CRP) and procalcitonin. ICU hemoglobin was lower in non-survivors, and serum albumin was lower at the margin of statistical significance (*p* = 0.0496). Neither PIV score differed between the outcome groups, and the baseline PILE score did not differ. The ICU-admission PILE score was slightly higher in patients who died (*p* = 0.037), a difference well below that seen for the severity scores (Table 2).

**Table 2:** Continuous variables at diagnosis and at ICU admission, by ICU mortality.

Variable	Died (*n* = 54)	Discharged (*n* = 21)	*p*
APACHE-II, mean ± SD	29.3 ± 7.1	22.6 ± 5.4	**<0**.**001**
SOFA, mean ± SD	9.8 ± 2.9	7.0 ± 2.6	**<0**.**001**
Diagnosis LDH, U/L, median (IQR)	267 (194–477)	208 (166–245)	**0**.**021**
ICU hemoglobin, g/dL, mean ± SD	7.3 ± 1.2	8.1 ± 1.2	**0**.**017**
ICU LDH, U/L, median (IQR)	354 (234–890)	229 (187–360)	**0**.**024**
ICU CRP, mg/L, mean ± SD	209.9 ± 98.8	168.4 ± 70.8	**0**.**048**
ICU procalcitonin, µg/L, median (IQR)	2.5 (0.8–11.3)	0.6 (0.4–1.9)	**0**.**001**
ICU albumin, g/L, mean ± SD	26.0 ± 4.8	28.6 ± 5.0	**0**.**0496**
Baseline PIV, median (IQR)	46.6 (3.5–553)	40.0 (1.4–240)	0.633
ICU PIV, median (IQR)	9.7 (0.4–326)	2.4 (0.2–33)	0.254
Baseline PILE, median (IQR)	2 (1–3)	1 (1–2)	0.103
ICU PILE, median (IQR)	2 (2–3)	2 (1–3)	**0**.**037**

CRP, C-reactive protein; LDH, lactate dehydrogenase; PIV, pan-immune-inflammation value; PILE, PIV + LDH + ECOG score.

Groups compared by the independent-samples *t*-test (mean ± SD) or the Mann–Whitney *U*-test [median (IQR)]. Vasopressor use and other events during the ICU stay are shown separately (Table 3). *p*-values below 0.05 are shown in bold.

During the ICU stay, infectious and organ complications were common. Pneumonia occurred in 85.3% of patients, sepsis in 76.0%, and acute kidney injury in 61.3%. Mechanical ventilation was required in 76.0% of patients, of whom 7% were successfully extubated, and vasopressors were administered in 76.0% for a median duration of 4 days. Soft-tissue infection occurred in 18.7% (Table 3). The median ICU length of stay was 5 days (IQR 3–10), with no statistically significant difference between those who died and those discharged alive (*p* = 0.32). Furthermore, none of the six scores correlated with length of stay, the closest being APACHE-II (rho −0.21; *p* = 0.071).

**Table 3:** Events during the ICU stay (*n* = 75).

Event	*n* (%)
Pneumonia	64 (85.3)
Sepsis	57 (76.0)
Acute kidney injury	46 (61.3)
Soft-tissue infection	14 (18.7)
Invasive mechanical ventilation	57 (76.0)
Extubated	4/57 (7.0)
Vasopressor support	57 (76.0)
Vasopressor days, median (IQR)^a^	4 (2–5)
ICU stay, days, median (IQR)	5 (3–10)

a Among the 57 patients who received vasopressors. Extubation is expressed as a proportion of the intubated patients.

In ROC analysis, APACHE-II and SOFA discriminated ICU mortality effectively (AUC 0.778, 95% CI 0.646–0.892; and 0.758, 0.640–0.865). Neither PIV score nor the baseline PILE score discriminated ICU mortality (all *p* > 0.05), and the ICU-admission PILE score showed only weak discrimination (AUC 0.647; *p* = 0.037), well below that of the established ICU severity scores. On pairwise DeLong testing, the two severity scores were comparable (*p* = 0.748). Both discriminated significantly better than either PIV score, and SOFA also discriminated better than the baseline PILE score (*p* = 0.035), only the comparisons with the ICU-admission PILE score did not reach statistical significance (APACHE-II, *p* = 0.200; SOFA, *p* = 0.234). Eight comparisons were made between a severity score and an inflammatory index, and after Bonferroni correction (*p* < 0.00625) the difference between SOFA and the baseline PIV score remained statistically significant (Table 4, Figure 2, Supplementary Table S1). The other count-based inflammatory indices in common use behaved similarly. Neither the neutrophil-to-lymphocyte, platelet-to-lymphocyte, nor monocyte-to-lymphocyte ratio discriminated ICU mortality at either time point, nor did the systemic immune-inflammation index or the systemic inflammation response index. The C-reactive protein-to-albumin ratio was the only parameter to reach nominal significance (AUC 0.660; *p* = 0.032), which did not survive correction for multiple testing across the 11 comparisons (Supplementary Table S2).

**Table 4:** Discrimination of ICU mortality.

Score	AUC (95% CI)	*p*	Cut-off	Sensitivity	Specificity
APACHE-II	0.778 (0.646–0.892)	**<0**.**001**	25	0.76	0.76
SOFA	0.758 (0.640–0.865)	**0**.**001**	8	0.78	0.62
Baseline PIV	0.536 (0.387–0.682)	0.633	—	—	—
ICU PIV	0.586 (0.444–0.718)	0.254	—	—	—
Baseline PILE	0.616 (0.484–0.746)	0.103	—	—	—
ICU PILE	0.647 (0.508–0.782)	**0**.**037**	2	0.76	0.48

AUC, area under the curve; PIV, pan-immune-inflammation value; PILE, PIV + LDH + ECOG score.

Cut-off by the Youden index, shown only for scores that discriminated (*p* < 0.05). Areas under the curve were compa*r*ed pairwise by the DeLong test; all 15 comparisons are given in Supplementary Table S1. *p*-values below 0.05 are shown in bold.

**Figure 2:**
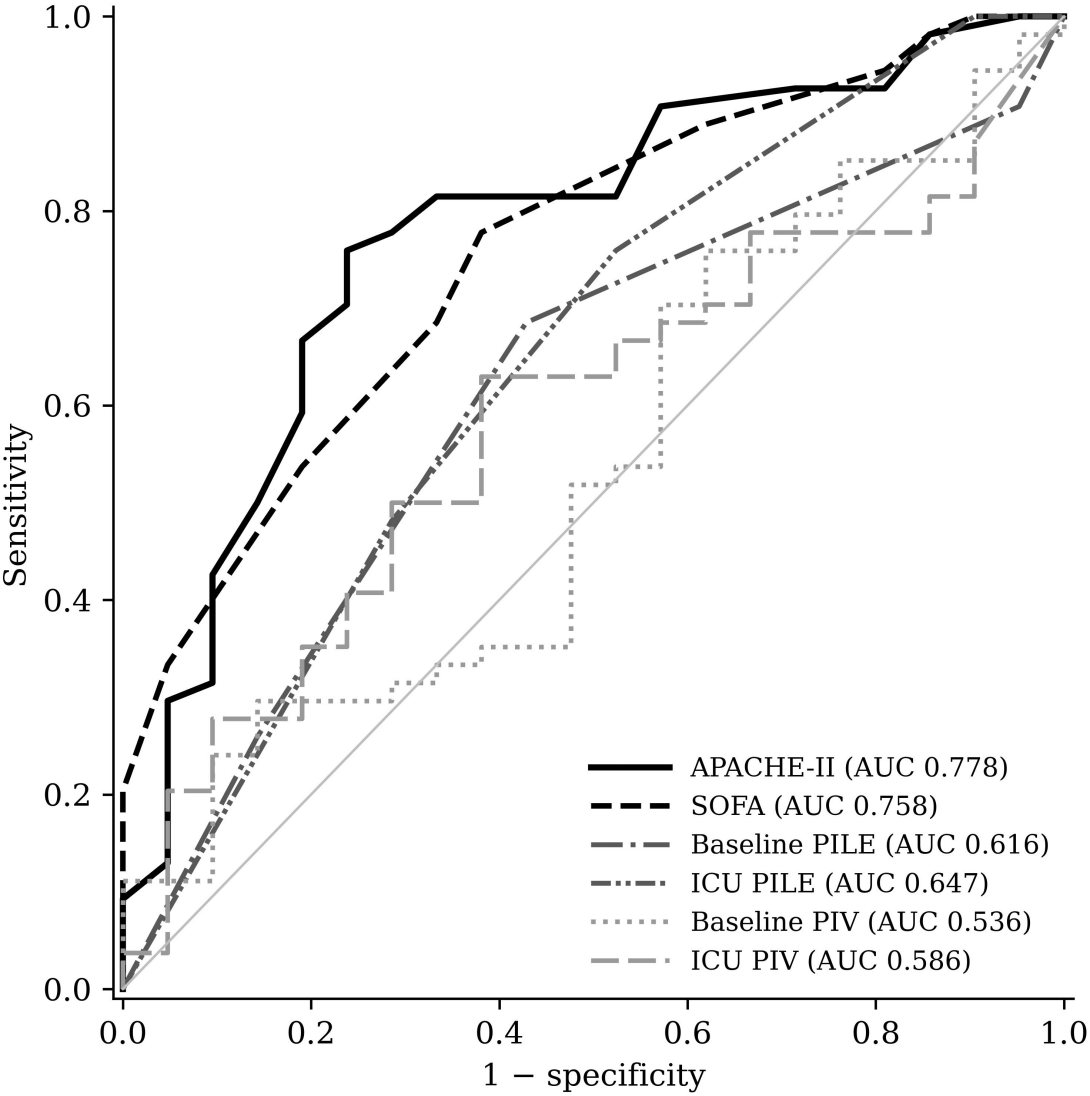
Receiver operating characteristic curves for the prediction of ICU mortality. APACHE-II and SOFA were assessed as continuous scores. AUC, area under the curve; PIV, pan-immune-inflammation value; PILE, PIV + LDH + ECOG score.

To test whether PIV or PILE added prognostic information beyond the established scores, each was entered alongside APACHE-II and SOFA in nested logistic regression models for ICU mortality. The baseline PILE score added little discriminative value and did not reach statistical significance over either score (APACHE-II, ΔAUC +0.014, likelihood ratio *p* = 0.31; SOFA, ΔAUC −0.001, *p* = 0.51). The ICU-admission PILE score showed a small increment that was nominally significant over APACHE-II (ΔAUC +0.043, *p* = 0.043) but not over SOFA (ΔAUC +0.023, *p* = 0.061). The bootstrap confidence interval for this increment crossed zero, and the *p*-value did not meet the threshold corrected for the four comparisons (*p* < 0.0125) (Table 5). PIV added no discrimination over APACHE-II at either time point (ICU PIV, ΔAUC +0.010, *p* = 0.48).

**Table 5:** Incremental value of the PILE score over established severity scores for ICU mortality.

Base model	Added score	Base AUC	Combined AUC	ΔAUC (95% CI)	LR *p*
APACHE-II	+ baseline PILE	0.778	0.792	+0.014 (−0.006 to +0.081)	0.31
APACHE-II	+ ICU PILE	0.778	0.821	+0.043 (−0.003 to +0.144)	**0**.**043**
SOFA	+ baseline PILE	0.758	0.757	−0.001 (−0.014 to +0.062)	0.51
SOFA	+ ICU PILE	0.758	0.782	+0.023 (−0.009 to +0.132)	0.061

AUC, area under the curve; LR, likelihood ratio; PIV, pan-immune-inflammation value; PILE, PIV + LDH + ECOG score.

Each PILE score was added to the severity score in a nested logistic regression model. ΔAUC 95% CI by bootstrap (3,000 resamples); LR *p* by likelihood ratio test comparing the nested models. With four comparisons, the Bonferroni-corrected significance threshold is *p* < 0.0125. *p*-values below 0.05 are shown in bold.

Over the whole follow-up, 72 of 75 patients died. Survival was analyzed from diagnosis with delayed entry at ICU admission, which removed the guaranteed survival time that would otherwise inflate the estimates (the interval from diagnosis to ICU admission was a median of 70 days). Measured from ICU admission, median survival was 7 days (IQR 3–27), and 76% of patients had died within 30 days. On univariable delayed-entry Cox regression, APACHE-II, SOFA, and both the baseline and ICU PILE scores were associated with overall survival, whereas neither PIV score was (Table 6). The baseline PILE association held after adjustment for ELN risk, comorbidity, induction treatment, and disease status (hazard ratio, HR, 1.95, 95% CI 1.33–2.85; *p* = 0.001). It did not carry over to the shorter time frame that begins at ICU admission, where the adjusted estimate fell to 1.33 (0.99–1.78; *p* = 0.060), and the risk groups differed only marginally (Figure 3).

**Table 6:** Association of the scores with overall survival.

Score	HR (95% CI)	*p*
Univariable analysis (delayed-entry Cox regression)		
APACHE-II (per point)	1.13 (1.09–1.18)	**<0**.**001**
SOFA (per point)	1.23 (1.13–1.35)	**<0**.**001**
Baseline PILE (per point)	1.66 (1.23–2.24)	**<0**.**001**
ICU PILE (per point)	1.49 (1.14–1.95)	**0**.**004**
Baseline PIV (per SD, log)	1.24 (0.95–1.62)	0.110
ICU PIV (per SD, log)	1.05 (0.82–1.36)	0.681
Baseline PILE, delayed entry from diagnosis		
Adjusted (per point)^a^	1.95 (1.33–2.85)	**0**.**001**
High risk (≥2) vs. low	1.90 (1.16–3.11)	**0**.**010**
Baseline PILE, survival measured from ICU admission		
Unadjusted (per point)	1.24 (0.95–1.62)	0.113
Adjusted (per point)^a^	1.33 (0.99–1.78)	0.060
High risk (≥2) vs. low	1.54 (0.96–2.48)	0.076

HR, hazard ratio; PIV, pan-immune-inflammation value; PILE, PIV + LDH + ECOG score.

a Adjusted for ELN 2022 adverse risk, Charlson comorbidity index, intensive induction chemotherapy, and active disease at ICU admission. Delayed-entry models use time from diagnosis with entry into the risk set at ICU admission; 72 of 75 patients died. PIV was log-transformed after adding a constant equal to half the smallest positive value observed at that time point, since neutrophil or monocyte counts of zero gave a PIV of zero in nine patients at ICU admission; hazard ratios are per standard deviation of the transformed value. *p*-values below 0.05 are shown in bold.

**Figure 3:**
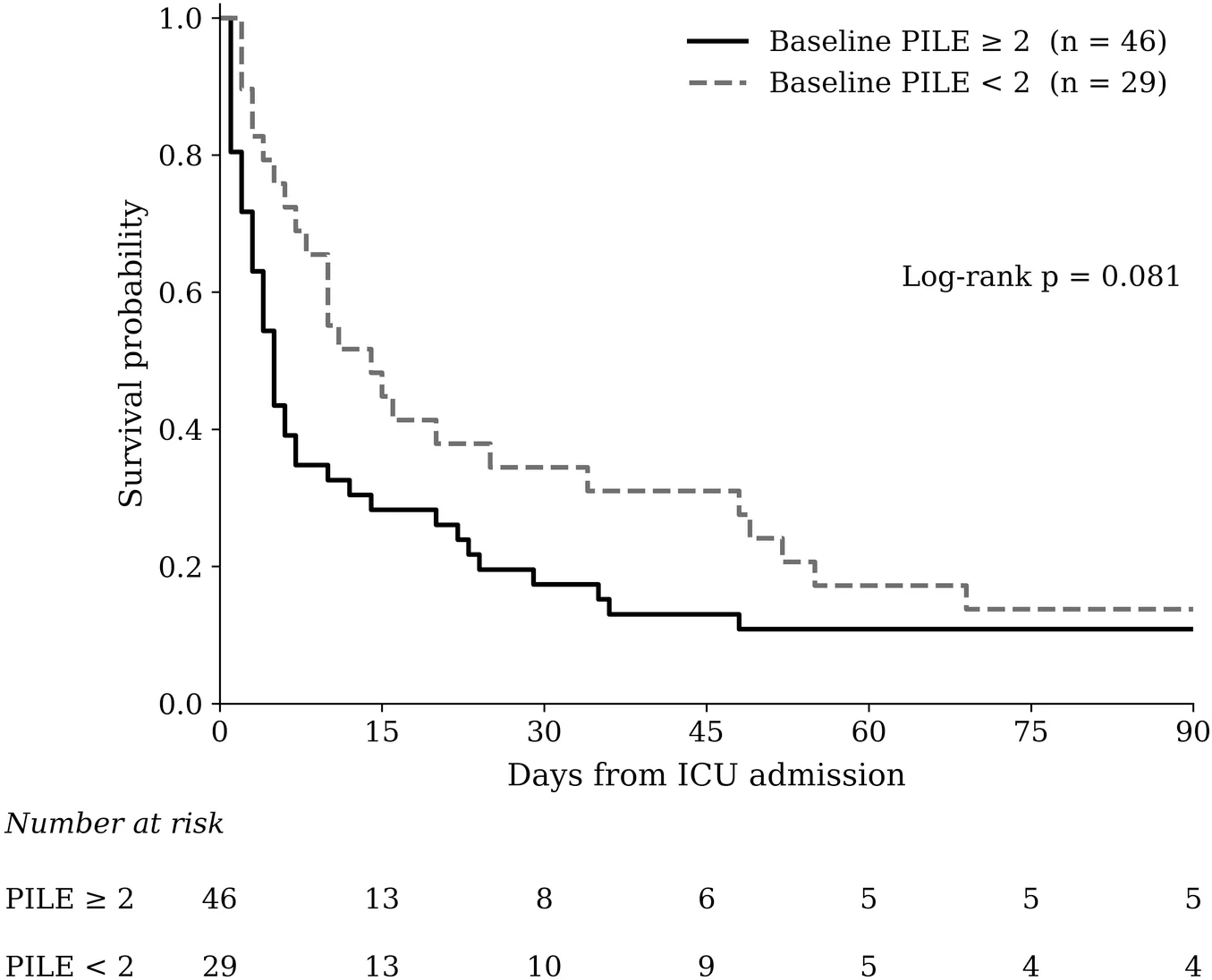
Survival from ICU admission by baseline PILE risk group. The comparison is unadjusted; adjusted estimates and the delayed-entry analysis measured from diagnosis are given in Table 6. PILE, PIV + LDH + ECOG score.

Several sensitivity analyses were performed to determine whether the findings depended on how the indices had been categorized. When PIV was split at its own time-point median, ICU mortality was similar in the high and low groups at diagnosis (73.7% vs. 70.3%, *p* = 0.74) and at ICU admission (78.9% vs. 64.9%, *p* = 0.17). Mortality was higher in both PILE high-risk groups (baseline, 80.4% vs. 58.6%, *p* = 0.040; ICU, 78.8% vs. 56.5%, *p* = 0.047); for the baseline score this contrasts with the continuous analysis, in which it did not discriminate ICU mortality. Recomputing both scores with a single pooled PIV threshold did not change the pattern (Supplementary Table S3).

Two further exploratory analyses examined the individual blood cell counts and the ELN risk groups. Of the counts measured at ICU admission, only hemoglobin differed between the outcome groups (*p* = 0.017) and discriminated ICU mortality, with lower values being associated with increased death risk (area under the curve 0.666, 95% CI 0.529–0.796). Platelet count did not discriminate mortality (0.515, 0.364–0.674), and none of the leukocyte subsets reached statistical significance (Supplementary Table S4). Within the intermediate-risk (*n* = 29) and adverse-risk (*n* = 40) groups, APACHE-II and SOFA discriminated ICU mortality, whereas neither PIV score nor PILE score demonstrated significant discrimination (Supplementary Table S5). One result changed when the baseline PILE score was split at its threshold: among intermediate-risk patients, mortality was 94% (16/17) in the high-risk group and 50% (6/12) in the low-risk group (*p* = 0.011). As a continuous variable the same score did not discriminate (area under the curve 0.711; *p* = 0.080), the smallest area this subgroup could have detected with 80% power was 0.764, and the comparison did not meet the Bonferroni-corrected threshold for the 16 subgroup tests (*p* < 0.0031).

## Discussion

In this cohort of 75 patients aged 65 years or older admitted to the intensive care unit with AML, almost three-quarters died in the unit, and neither PIV nor the PILE score identified them. The PILE score measured at ICU admission reached nominal significance, but its discrimination was modest and well below APACHE-II and SOFA, and once the four comparisons were accounted for via multiple-testing correction, adding either index to those scores provided no incremental predictive value. Survival outcomes varied substantially depending on the chosen time origin. Measured from the date of diagnosis, a higher baseline PILE score was associated with shorter overall survival, and the association remained robust after adjustment; when evaluated from ICU admission, however, this prognostic association was no longer observed. During the ICU stay, the patients who died were distinguished by infection and organ failure rather than by any systemic inflammatory index.

PIV and PILE were developed in ambulatory oncology cohorts, where the constituent cell counts track the balance between tumor burden and host immunity over months (13–15). Both have since been applied to hematological malignancies, including primary central nervous system lymphoma (16) and a mixed-malignancy cohort (17), and in each case the outcome was survival measured over months to years. Critical illness sits at the opposite end of that time spectrum. Studies of PIV in sepsis have reached conflicting conclusions. In adults with urosepsis, it was associated with 28-day mortality and improved discrimination of established severity scores (18), whereas in older critically ill patients with sepsis it did not predict 28-day mortality (area under the curve 0.550) (19). Our results agree with the second, and our patients' counts were far more depleted: the median PIV in that older cohort was 867, more than 100 times the median value of 7.9 observed in our cohort. Whether a patient survives to ICU discharge is determined by the trajectory of acute organ failure over days, and the counts drawn on the morning of admission no longer read as a signal from the leukemia alone; they carry the imprint of recent chemotherapy, bone marrow aplasia, blood product transfusion, and sepsis. The problem also lies in how the index is formulated. In nine of our patients the neutrophil or monocyte count was zero at ICU admission, which drove PIV to zero and placed them below the cohort median, so the score treated the most complete bone marrow failure in the series as the lowest inflammatory risk. An index calculated as the product of cellular counts has limited ability to stratify cytopenic patients.

The conceptual limitation extends beyond a zero cell count. PIV multiplies four cell populations as though each contributed to risk in a fixed direction and with a fixed biological significance, but the cells involved function at different stages of a single immune response. Early in that response, neutrophils are recruited to tissue; as it resolves, recruitment stops, and macrophages clear the apoptotic neutrophils (20). A given neutrophil or monocyte count can therefore reflect an early, an evolving, or a resolving phase, which cannot be distinguished by static cellular ratios alone. Furthermore, the index name implies a separation between immunity and inflammation that its arithmetic does not capture, since inflammation is one phase of the immune response rather than a process apart from it. Our data does not allow the specific phase of the response at ICU admission to be identified, and none of the count-based indices we examined accounts for these temporal dynamics. In a patient whose bone marrow has failed, the product of the counts falls to zero, and the construct breaks down altogether, the extreme case of a limitation present in every patient.

APACHE-II and SOFA were the strongest predictors of ICU mortality in our cohort, and we modeled both as continuous variables instead of dichotomizing them at a threshold. Each was designed to quantify acute physiological derangement and organ dysfunction (9, 10), which principally determine the outcome of an intensive care admission. In pairwise DeLong testing, the two scores demonstrated comparable discrimination, and each discriminated significantly better than either PIV score; only comparisons with the ICU-admission PILE score fell short of significance. Adding PIV or PILE to either severity score left discrimination largely unchanged, and the one nominally significant increment did not hold up when all four comparisons were taken into account. Earlier work in critically ill AML points the same way. Schellongowski et al. found SAPS II to be the only independent predictor of survival among patients with AML who required intensive care (7). In a larger series SOFA discriminated ICU mortality better than SAPS II or the Logistic Organ Dysfunction score, although a disease-specific score outperformed all three (12), and a higher SOFA score was likewise associated with mortality in a smaller cohort (21). Survival in our series was lower than in those reports, which reflects the inherent risk profile of a cohort restricted to patients aged 65 years and older, all of whom were critically ill. Scores that track acute organ failure fit the reality of the unit, where early death is driven by physiological collapse rather than by leukemia biology alone.

Whether a severity score performs consistently across etiologies of admission is a salient question, and the evidence suggests it does not. SOFA was revised as SOFA-2 after our data were collected (22). In a large external validation of the revised score, discrimination of 30-day mortality was an AUC of 0.81 in patients admitted after trauma but 0.72 in those with sepsis, who were older and had higher comorbidity burdens (23). Our patients were older and more comorbid still, and most were septic. SOFA discriminated ICU mortality in our cohort, with an AUC of 0.758, although a different endpoint and a far smaller sample size make these estimates only loosely comparable. A score validated across the general ICU population therefore requires testing in each specific population in which it is used, and evaluations confined to one disease, such as ours, provide essential disease-specific validation.

None of the baseline clinical characteristics discriminated between patients who died and those who survived, and serum LDH was the only biomarker measured at diagnosis that did. Most of what distinguished the two groups was the state of infection and organ function on the day they arrived. Procalcitonin carried the clearest signal, with LDH and C-reactive protein behind it. Hemoglobin concentration was lower in non-survivors, and serum albumin was lower at the margin of significance. The failure of PIV and PILE was not peculiar to those two scores. None of the other count-based indices in common use discriminated ICU mortality at either time point, and the only marker with any discriminative capacity was the C-reactive protein-to-albumin ratio, which reflects the acute-phase response and nutritional status instead of peripheral cell count and has been reported as an outcome marker in critically ill patients (24). That association was nominal and did not survive multiple-testing correction, so it belongs in the same category as the rest: a lead, not a finding. Infection was pervasive across the cohort, with pneumonia in more than four-fifths of patients and sepsis and acute kidney injury in the majority. We deliberately kept these events out of the predictive analysis, because they develop during the stay and are shaped by it; treating them as baseline markers would read a consequence as a cause (25). What the admission values support is a narrower and safer claim: at the moment a patient with AML reaches intensive care, what distinguishes outcomes is the acute infectious and physiological insult, not the inflammatory arithmetic of the blood cell count.

On the time scale from diagnosis, with each patient entering the analysis at the time of ICU admission, baseline PILE was associated with overall survival, and adjustment for ELN risk, comorbidities, induction treatment, and disease status did not eliminate this association. This fits what the score is assembled from. LDH tracks tumor burden and tissue turnover and has been linked to mortality in AML (26); performance status remains among the most durable prognostic factors in older patients with the disease (3, 27); and the inflammatory component adds a reading of host state (14). The same models, though, put APACHE-II and SOFA in the same position, so whatever PILE carries on this time scale is not unique to it. When counted from ICU admission, the association fell away, and the adjusted estimate was no longer significant.

The clinical implication of this pattern is worth stating plainly. When almost three-quarters of a cohort dies in the unit, the outcome is dominated by the acute insult, and that insult is measured directly, and better, by physiological scores; an index assembled from blood counts is left with little to add on the day of admission. High mortality of this kind narrows what any bedside marker can contribute during the ICU stay. It does not, however, make risk stratification pointless in these patients. It moves the optimal window for evaluation earlier. If the burden of leukemia and the patient's fitness are to inform how aggressively an older patient is treated, and how early intensive care is discussed, the time to measure them is at diagnosis, before the deterioration that brings the patient to the ICU, rather than on the day of arrival. Clinicians in the ICU already weigh proportionality: in a multinational cohort, patients with uncontrolled cancer were more often perceived by ICU clinicians as receiving excessive care than patients with controlled cancer or without cancer, even after adjustment for case mix (28). An objective risk assessment taken at diagnosis could bring that judgment forward.

Several features of the design represent distinct methodological strengths. To our knowledge, this study is the first to evaluate PIV and PILE in older patients with AML during critical illness, and the first to compare their discriminative performance directly with that of APACHE-II and SOFA for ICU mortality. PIV has been associated with survival in AML previously, but on the ward and over a horizon of months (17), and PILE has been studied mainly in lymphoma (16); neither had been examined where the immediate clinical endpoint is whether the patient survives to ICU discharge. Computing both scores at diagnosis and again at ICU admission separated the baseline biology of the leukemia from the acute illness that brought the patient in. For survival, we used delayed entry, so that the time lived between diagnosis and admission was not artificially incorporated into the cohort follow-up, and we tested the incremental value of the indices in nested logistic regression models, against the severity scores instead of comparing separate univariate regressions. The scores were also benchmarked against a wider panel of count-based indices, and the PILE association with survival was adjusted for the main clinical confounders, not for disease status alone. Reporting the negative and exploratory results alongside the isolated nominally positive signal provides an unbiased, balanced assessment of index utility in this high-acuity setting.

### Limitations

This study has several important limitations. It was retrospective and conducted at a single center, and the referral pattern of our ICU may not be representative of that of others. No independent cohort was available to validate the findings, and until they are reproduced elsewhere, they describe this population and should not be read as generalizable. The sample of 75 patients is small, and the confidence intervals around the areas under the curve are correspondingly wide. A nonsignificant result in a cohort of this size does not establish that PIV and PILE are entirely devoid of prognostic value (29). With 54 deaths and 21 survivors, the smallest area under the curve separable from 0.5 with 80% power was 0.681, so the discrimination observed for either PIV score was below what this sample could have detected, while the areas of 0.778 and 0.758 for APACHE-II and SOFA were detected with more than 99% statistical power. For overall survival, the study was better powered: with 72 deaths, a hazard ratio of 1.39 per standard deviation was detectable, and the associations we report exceed that threshold. The incremental analyses were the least well powered, since a difference of 0.286 in the area under the curve would have been needed, far larger than any increment these indices plausibly provide. The negative result for ICU mortality is therefore firmer for PIV than for the ICU-admission PILE score, and the incremental analyses should be interpreted as underpowered to detect a small prognostic contribution rather than as excluding one conclusively.

The area under the curve has inherent limitations of its own as a summary of discriminative performance. It averages discrimination over every possible cut-off, including those of no clinical utility, takes no account of outcome prevalence or the different costs of false-positive and false-negative results, and a change in its value carries little direct clinical meaning (30). Our incremental analyses rest on such changes, and the areas we report describe discrimination in this specific cohort and may not be directly generalizable to other patient populations. The PIV thresholds were derived from the medians of our own cohort, which ties them to this sample and limits the external transportability of these specific cutoffs. Splitting a continuous index at any threshold also discards information and introduces classification error near the boundary (31, 32), which is why PIV was modeled as a continuous variable in the regression models and why the analyses were repeated with a pooled cut-off. The baseline PILE score exemplifies the pitfalls of threshold categorization. In the whole cohort, it separated the risk groups when dichotomized but did not discriminate ICU mortality as a continuous score. The same discrepancy appeared among intermediate-risk patients, where the dichotomized score yielded a mortality of 94% against 50% while the continuous score fell short of statistical significance.

The date of each Eastern Cooperative Oncology Group (ECOG) performance status assessment was not recorded, so the interval between the documented score and ICU admission varied between patients and could not be quantified; a score documented several months earlier may no longer have reflected the functional status of the patient upon arrival. Delayed entry corrects survival time for the period lived before the cohort was assembled, but it cannot correct for the confounding by indication or selection mechanisms that decided which patients reached intensive care at all, and that decision was inherently shaped by prognosis. The supplementary analyses were exploratory and should be interpreted as such. Whether PILE has any clinical role in this population will require larger, multicenter prospective studies to determine conclusively.

## Conclusion

In this single-center cohort of critically ill older patients with AML, neither PIV nor PILE predicted ICU mortality. Although the cohort was better powered to exclude a prognostic role for PIV than for the ICU-admission PILE score, adding either index to APACHE-II or SOFA did not improve on what those scores already provided. For overall survival measured from diagnosis, baseline PILE remained statistically significant after multivariable adjustment, though the finding was not specific to it, and the association did not persist when survival was measured from the day of admission. In this setting, acute physiological severity was the better guide to outcome. If leukemia-derived inflammatory indices have clinical utility in this population, their value is more likely to be realized at diagnosis, while treatment intensity and the ceiling of care remain open questions, and that possibility needs testing in larger multicenter cohorts.

## Data Availability

The raw data supporting the conclusions of this article will be made available by the authors, without undue reservation.
