# Supplementary material for: Neither the pan-immune-inflammation value nor the PILE score improved on APACHE-II or SOFA in a single-center cohort of critically ill older patients with acute myeloid leukemia
Source: Front Med (Lausanne). 2026 Oct 27;13:1955731. doi: 10.3389/fmed.2026.1955731 (PMC13748412; doi:10.3389/fmed.2026.1955731)
Supplement: Supplementary file 1 [file Table1.docx]

**Supplementary Table S1. Pairwise comparison of the areas under the curve for ICU mortality.**

| **Comparison** | **AUC** | **ΔAUC** | **p** |
| --- | --- | --- | --- |
| ***Severity score versus inflammatory index*** | | | |
| SOFA vs baseline PIV | 0.758 vs 0.536 | +0.222 | **0.001** |
| APACHE-II vs baseline PIV | 0.778 vs 0.536 | +0.242 | **0.007** |
| SOFA vs baseline PILE | 0.758 vs 0.616 | +0.142 | **0.035** |
| APACHE-II vs ICU PIV | 0.778 vs 0.586 | +0.192 | **0.039** |
| SOFA vs ICU PIV | 0.758 vs 0.586 | +0.173 | **0.046** |
| APACHE-II vs baseline PILE | 0.778 vs 0.616 | +0.162 | 0.079 |
| APACHE-II vs ICU PILE | 0.778 vs 0.647 | +0.131 | 0.200 |
| SOFA vs ICU PILE | 0.758 vs 0.647 | +0.112 | 0.234 |
| ***Between the two severity scores*** | | | |
| APACHE-II vs SOFA | 0.778 vs 0.758 | +0.019 | 0.748 |
| ***Between inflammatory indices*** | | | |
| Baseline PILE vs baseline PIV | 0.616 vs 0.536 | +0.080 | 0.143 |
| ICU PILE vs baseline PIV | 0.647 vs 0.536 | +0.111 | 0.200 |
| ICU PILE vs ICU PIV | 0.647 vs 0.586 | +0.061 | 0.256 |
| Baseline PIV vs ICU PIV | 0.536 vs 0.586 | −0.049 | 0.565 |
| Baseline PILE vs ICU PILE | 0.616 vs 0.647 | −0.031 | 0.700 |
| Baseline PILE vs ICU PIV | 0.616 vs 0.586 | +0.030 | 0.704 |
| *All fifteen pairwise comparisons of the areas under the curve for ICU mortality, by the DeLong test, ordered by p value within each block. ΔAUC is the difference between the first and the second score of each pair. Eight comparisons address the question of whether a severity score discriminates better than an inflammatory index; with that number of comparisons the Bonferroni-corrected threshold is p < 0.00625. AUC, area under the curve; PIV, pan-immune-inflammation value; PILE, PIV + LDH + ECOG score. p values below 0.05 are shown in bold.* | | | |
