# Supplementary material for: Neither the pan-immune-inflammation value nor the PILE score improved on APACHE-II or SOFA in a single-center cohort of critically ill older patients with acute myeloid leukemia
Source: Front Med (Lausanne). 2026 Oct 27;13:1955731. doi: 10.3389/fmed.2026.1955731 (PMC13748412; doi:10.3389/fmed.2026.1955731)
Supplement: Supplementary file 2 [file Table2.docx]

**Supplementary Table S2. Discrimination of ICU mortality by other count-based inflammatory indices.**

| **Index** | **AUC (95% CI)** | **p** |
| --- | --- | --- |
| ***At diagnosis*** | | |
| Neutrophil-to-lymphocyte ratio | 0.528 (0.392–0.672) | 0.710 |
| Platelet-to-lymphocyte ratio | 0.372 (0.218–0.533) | 0.088 |
| Monocyte-to-lymphocyte ratio | 0.577 (0.422–0.727) | 0.305 |
| Systemic immune-inflammation index | 0.475 (0.330–0.625) | 0.746 |
| Systemic inflammation response index | 0.583 (0.421–0.729) | 0.267 |
| ***At ICU admission*** | | |
| Neutrophil-to-lymphocyte ratio | 0.525 (0.385–0.663) | 0.741 |
| Platelet-to-lymphocyte ratio | 0.436 (0.298–0.589) | 0.392 |
| Monocyte-to-lymphocyte ratio | 0.599 (0.439–0.743) | 0.186 |
| Systemic immune-inflammation index | 0.511 (0.369–0.656) | 0.892 |
| Systemic inflammation response index | 0.593 (0.454–0.732) | 0.213 |
| C-reactive protein-to-albumin ratio | 0.660 (0.529–0.781) | **0.032** |
| *All indices were analyzed for the whole cohort (n = 75). Confidence intervals are from 2000 bootstrap resamples and p values from the Mann–Whitney U test. These analyses were exploratory; with eleven comparisons a Bonferroni-corrected threshold of p < 0.0045 would apply, which none of the indices met. Ratios were computed from the neutrophil, lymphocyte, monocyte and platelet counts of the same sample; C-reactive protein and albumin were available only at ICU admission. AUC, area under the curve; ICU, intensive care unit. p values below 0.05 are shown in bold.* | | |
