# Supplementary material for: Neither the pan-immune-inflammation value nor the PILE score improved on APACHE-II or SOFA in a single-center cohort of critically ill older patients with acute myeloid leukemia
Source: Front Med (Lausanne). 2026 Oct 27;13:1955731. doi: 10.3389/fmed.2026.1955731 (PMC13748412; doi:10.3389/fmed.2026.1955731)
Supplement: Supplementary file 3 [file Table3.docx]

**Supplementary Table S3. Discrimination of ICU mortality when the PILE scores are computed with a single pooled PIV threshold instead of the time-point medians.**

| **Score** | **PIV threshold** | **High-risk, n** | **AUC** | **p** |
| --- | --- | --- | --- | --- |
| Baseline PILE | Own time-point median (46.25) | 46 | 0.616 | 0.103 |
|  | Pooled median (17.23) | 50 | 0.604 | 0.139 |
| ICU PILE | Own time-point median (7.89) | 52 | 0.647 | **0.037** |
|  | Pooled median (17.23) | 52 | 0.664 | **0.021** |
| *The pooled threshold was derived from the diagnosis and ICU-admission PIV values combined. Five patients at diagnosis and six at ICU admission moved to a different score. Groups compared by the Mann–Whitney U test. AUC, area under the curve; PIV, pan-immune-inflammation value; PILE, PIV + LDH + ECOG score. p values below 0.05 are shown in bold.* | | | | |
