# Supplementary material for: Neither the pan-immune-inflammation value nor the PILE score improved on APACHE-II or SOFA in a single-center cohort of critically ill older patients with acute myeloid leukemia
Source: Front Med (Lausanne). 2026 Oct 27;13:1955731. doi: 10.3389/fmed.2026.1955731 (PMC13748412; doi:10.3389/fmed.2026.1955731)
Supplement: Supplementary file 4 [file Table4.docx]

**Supplementary Table S4. Blood count parameters at ICU admission and their discrimination of ICU mortality.**

| **Parameter at ICU admission** | **Died (n = 54)** | **Discharged (n = 21)** | **p** | **AUC (95% CI)** |
| --- | --- | --- | --- | --- |
| Leukocytes, ×10⁹/L | 1.9 [0.3–10.4] | 0.7 [0.4–4.0] | 0.278 | 0.582 (0.450–0.716) |
| Neutrophils, ×10⁹/L | 0.6 [0.0–4.1] | 0.2 [0.1–0.6] | 0.539 | 0.546 (0.412–0.674) |
| Lymphocytes, ×10⁹/L | 0.5 [0.1–1.5] | 0.3 [0.1–0.6] | 0.235 | 0.589 (0.451–0.723) |
| Monocytes, ×10⁹/L | 0.4 [0.0–2.9] | 0.1 [0.0–0.7] | 0.162 | 0.605 (0.453–0.745) |
| Platelets, ×10⁹/L | 24 [13–36] | 21 [13–44] | 0.850 | 0.515 (0.364–0.674) |
| Hemoglobin, g/dL, mean ± SD | 7.3 ± 1.2 | 8.1 ± 1.2 | **0.017** | 0.666 (0.529–0.796)ᵃ |
| *Values are median [IQR] unless stated. Groups were compared by the Mann–Whitney U test or, for hemoglobin, the independent-samples t test. Areas under the curve are for discrimination of ICU mortality, with 95% confidence intervals from 3000 bootstrap resamples. p values below 0.05 are shown in bold. ᵃArea under the curve for lower values predicting death. AUC, area under the receiver operating characteristic curve; ICU, intensive care unit.* | | | | |
