# Supplementary material for: Neither the pan-immune-inflammation value nor the PILE score improved on APACHE-II or SOFA in a single-center cohort of critically ill older patients with acute myeloid leukemia
Source: Front Med (Lausanne). 2026 Oct 27;13:1955731. doi: 10.3389/fmed.2026.1955731 (PMC13748412; doi:10.3389/fmed.2026.1955731)
Supplement: Supplementary file 5 [file Table5.docx]

**Supplementary Table S5. Discrimination of ICU mortality within ELN 2022 risk groups.**

| **Score** | **Intermediate risk (n = 29)** | **p** | **Adverse risk (n = 40)** | **p** |
| --- | --- | --- | --- | --- |
| ***Discrimination of ICU mortality, AUC (95% CI)*** | | | | |
| APACHE-II | 0.760 (0.519–0.955) | **0.044** | 0.839 (0.686–0.953) | **0.001** |
| SOFA | 0.834 (0.667–0.962) | **0.009** | 0.743 (0.557–0.895) | **0.016** |
| Baseline PIV | 0.597 (0.315–0.855) | 0.469 | 0.548 (0.354–0.737) | 0.647 |
| ICU PIV | 0.597 (0.348–0.819) | 0.460 | 0.613 (0.422–0.799) | 0.268 |
| Baseline PILE | 0.711 (0.442–0.913) | 0.080 | 0.533 (0.354–0.704) | 0.745 |
| ICU PILE | 0.656 (0.377–0.907) | 0.203 | 0.647 (0.467–0.817) | 0.113 |
| ***ICU mortality by PILE risk group, deaths/n (%)*** | | | | |
| Baseline PILE ≥ 2 | 16/17 (94) | **0.011** | 17/24 (71) | 1.000 |
| Baseline PILE < 2 | 6/12 (50) |  | 11/16 (69) |  |
| ICU PILE ≥ 2 | 18/21 (86) | 0.068 | 20/27 (74) | 0.476 |
| ICU PILE < 2 | 4/8 (50) |  | 8/13 (62) |  |
| *Deaths: 22 in the intermediate-risk group and 28 in the adverse-risk group. The two favorable-risk patients were not analyzed separately. Areas under the curve are given with 95% confidence intervals from 3000 bootstrap resamples and p values from the Mann–Whitney U test; risk groups were compared by Fisher’s exact test. p values below 0.05 are shown in bold. Counting all sixteen subgroup comparisons, the Bonferroni-corrected threshold was p < 0.0031; only APACHE-II in the adverse-risk group met it. With 80% power at a two-sided alpha of 0.05, the smallest area under the curve separable from 0.5 was 0.764 in the intermediate-risk group and 0.729 in the adverse-risk group. These analyses are exploratory. AUC, area under the receiver operating characteristic curve; ELN, European LeukemiaNet; ICU, intensive care unit; PIV, pan-immune-inflammation value.* | | | | |
